# Analysis and Design of a CLC/N Compensated CC-Type WPT System with Compact and Low-Cost Receiver

**DOI:** 10.3390/s23020838

**Published:** 2023-01-11

**Authors:** Lin Yang, Shuai Jiang, Can Wang, Li Zhang

**Affiliations:** 1 College of Electronic and Electrical Engineering, Henan Normal University, Xinxiang 453007, China; 2 Henan Key Laboratory of Optoelectronic Sensing Integrated Application, Henan Normal University, Xinxiang 453007, China; 3 Academician Workstation of Electromagnetic Wave Engineering of Henan Province, Henan Normal University, Xinxiang 453007, China; 4 College of International Education, Henan Normal University, Xinxiang 453007, China; 5 College of Life Sciences, Henan Normal University, Xinxiang 453007, China

**Keywords:** wireless power transfer (WPT), constant current (CC), CLC/none (CLC/N), zero-phase-angle (ZPA), zero-voltage switching (ZVS)

## Abstract

Wireless power transfer (WPT) has been extensively studied by technicians for its advantages of safety, convenience and aesthetics. The load-independent constant current (CC) output is the focus of WPT research and has been initially applied in various fields, such as light-emitting diodes (LEDs) driving, CC charging of electric vehicles (EVs), etc. However, the existing CC-type WPT system has problems in that the output current is constrained by the loosely coupled transformer (LCT) parameters, the receiver is bulky, and the development cost is high. Therefore, this manuscript proposes a new CLC/None (CLC/N) compensated WPT system with a CC output function that eliminates the receiver-side compensation components, ensures the compactness of the receiver, and saves on production costs. The conditions for satisfying the CC output and zero-phase-angle (ZPA) operation of the proposed system are first discussed. Then, the detailed parameter design method is provided, and the characteristic that the output current is unconstrained by the LCT parameters is illustrated. In addition, the implementation of zero-voltage switching (ZVS) operation of the proposed system and the sensitivity of the changes of compensation components to the output current are analyzed in detail. Furthermore, to demonstrate the superiority of the proposed system, several other typical CC-type WPT systems are introduced for comparison. Finally, a confirmatory experimental prototype with an output current of 2 A is fabricated, and the experimental results are consistent with the theoretical analysis.

## 1. Introduction

In recent years, wireless power transfer (WPT) has attracted much attention due to its advantages of safety, reliability, electrical isolation, and aesthetics [1]. WPT is a technology that converts electrical energy into other forms of energy and then into electrical energy to power electrical equipment, which mainly includes electromagnetic coupling [2], microwave [3], laser [4] and electric field coupling [5], etc. Due to low failure rate, high efficiency and mature development, WPT based on electromagnetic coupling has been widely applied in practical applications such as portable electronic devices [6], implantable biomedical products [7], underwater charging equipment [8], electric vehicles (EVs) [9,10], constant current (CC) source for LEDs driving [11] and other industrial fields [12,13].

At present, the key point of WPT research mainly includes compensation topology design [14,15], efficiency improvement scheme [16,17], anti-misalignment magnetic coupler design [18,19] and long-distance charging [20,21]. Among the above research, compensation topology design plays a crucial role in the power transfer efficiency and output performance of the WPT system. In previous work on WPT, most researchers tend to focus on the constant voltage (CV) output characteristic. However, in some specific WPT applications, the CC output characteristic is more desirable. The authors in [22] demonstrate that SS compensation topology can achieve a load-independent CC output function. However, the output current of the SS topology is constrained by the LCT parameters [23]. In other words, if the output current needs to be changed to match different application requirements during the system design stage, a new LCT has to be replaced, which undoubtedly reduces its compatibility. The authors in [23] propose a WPT system based on LC/CC topology, which can well realize CC characteristics that are not constrained by the LCT parameters. However, due to the parallel compensation capacitor on the receiver, a bulky filter inductor has to be added behind the rectifier, which inevitably increases the volume/weight and cost of the receiver. The CC-type WPT system based on LCL/P topology proposed in [24] also faces the same intractable problem as the CC-type WPT system in [23]. Although the CC-type WPT system based on LCC/LCC topology in [25] removes the filter inductor behind the rectifier, a large number of compensation components on both transmitter and receiver also increase the weight/volume and cost of the system. The four-coil structure WPT system with CC characteristic proposed in [26] has the same structural issue as the CC-type WPT system in [25]. The CC-type WPT system based on LC/CL compensation topology proposed in [27] reasonably reduces the number of compensation components. However, due to the existence of a bulky series compensation inductor on the receiver, the LCC/LCC topology, four-coil structure and LC/CL topology still cannot ensure the lightless and compactness of the receiver. In view of this, the three-coil structure WPT system with CC output function proposed in [28] can greatly improve the weight/volume and cost of the receiver because there is only one compensation capacitor on the receiver. Moreover, the CC-type WPT system based on LC/S topology proposed in [29] further reduces the number of compensation components on both the transmitter and receiver and maintains the compactness and low cost of the receiver. Although the WPT system based on LC/S topology is highly recognized for its structural and functional characteristics, there is still an unavoidable compensation capacitor on the receiver. To further improve the compactness and reduce the cost of the receiver of the WPT system, the authors in [30] propose a novel transmitter-side linear control technique to enable the LCC-N compensated WPT system to realize constant charging output. Although the compensation capacitor is eliminated on the receiver of the system, the DC-DC converter is additionally introduced in the front stage of the high-frequency inverter (HFI), which undoubtedly increases the hardware cost, weight and loss of the system. In addition, this method based on complex control technology greatly increases the difficulty of system controller design. Inspired by the research in [30], a new CLC/N compensated CC-type WPT system is proposed in this manuscript. The proposed system can achieve the load-independent CC output characteristic and ZPA operation through its own inherent structural attributes, and complicated control techniques are no longer necessary. In addition, the output current of the proposed system is unconstrained by the LCT parameters and has good compatibility. Furthermore, there are no compensation components and filter inductor on the receiver, which further reduces the weight/volume and cost, and ensures the compact and low-cost receiver.

The remainder of this manuscript is summarized as follows: In Section 2, the load-independent CC output characteristic of the proposed CLC/N compensated WPT system and the associated ZPA operation are discussed through theoretical analysis. Then, the detailed parameters tuning method of the CLC/N compensated WPT system is described in Section 3, and the load-independent CC output characteristics and ZPA operation are verified by simulation. In addition, to reduce the switching losses of MOSFETs in the HFI, the implementation conditions for ZVS operation and the sensitivity of the changes of compensation components to the output current are analyzed and discussed in detail. Furthermore, the comparison analysis between the proposed CLC/N compensated WPT system and the CLC/S compensated WPT system in terms of power transfer efficiency in Section 4 to reflect the advantages of secondary-side none compensation. In Section 5, an experimental prototype with an output current of 2 A is established to fully verify the feasibility of the proposed CLC/N compensated WPT system in terms of CC output characteristic, ZPA operation, and ZVS operation. Moreover, several other typical CC-type WPT systems are introduced for comparison to more intuitively reflect the advantages of the proposed CLC/N compensated WPT system in the functional and structural properties. Finally, conclusions are drawn in Section 6.

## 2. Theoretical Analysis

### 2.1. Overview of the CLC/N Compensated WPT System

**Figure 1:**
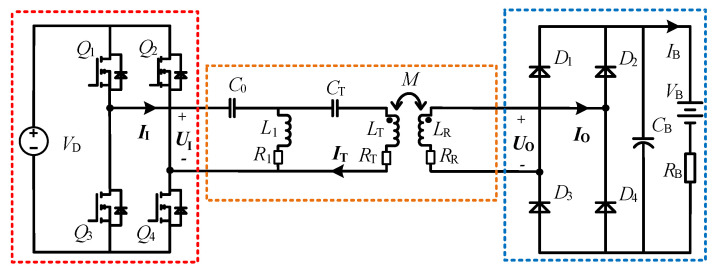
Architecture diagram of the CLC/N compensated WPT system.

The architecture diagram of the proposed CLC/N compensated WPT system is shown in Figure 1. $V_{D}$ is the DC input voltage source of the system, which drives the HFI consisting of four MOSFETs ($Q_{1}-Q_{4}$) to supply power for the transmitter. The operating frequency of the system is *f*, and its mathematical relationship to the angular frequency ω is expressed as ω=2πf. $L_{1}$, $L_{T}$ and $L_{R}$ are the parallel compensation inductance, self-inductances of transmitter-side and receiver-side coils, respectively, and $R_{1}$, $R_{T}$ and $R_{R}$ are the corresponding internal resistances.

*M* is mutual inductance between transceiver-side coils. A rectifier consisting of four diodes ($D_{1}-D_{4}$) is installed on the receiver, which powers the load $R_{B}$ by converting AC current into DC current. A filter capacitor $C_{B}$ is introduced behind the rectifier to stabilize the output. To facilitate the circuit analysis, the fundamental harmonic approximation (FHA) is adopted for the steady-state analysis of the resonant circuit. The AC equivalent resistance of the part surrounded by blue dotted lines in Figure 1 is expressed as $R_{O}$, which is deduced as [31]

(1) $$R_{O}=\frac{8}{\pi^{2}}R_{B}$$

**Figure 2:**
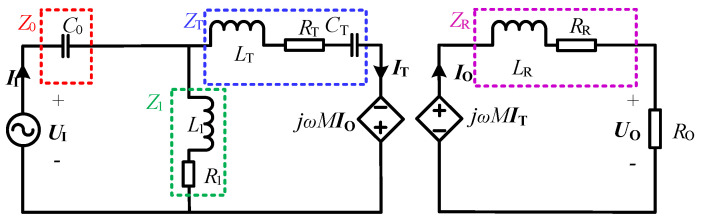
Equivalent circuit diagram of the CLC/N compensated WPT system.

The equivalent circuit diagram of the CLC/N compensated WPT system is shown in Figure 2. $U_{I}$ is the high-frequency AC voltage, and the root mean square (RMS) value is given as [31]

(2) $$U_{I}=\frac{2\sqrt{2}}{\pi }V_{D}$$

In addition, the mathematical functional relation between the system’s output current $I_{B}$ and the RMS value of the rectifier’s input current $I_{O}$ can be expressed as [31]

(3) $$I_{B}=\frac{2\sqrt{2}}{\pi }I_{O}$$

Based on Kirchhoff’s voltage law (KVL), the matrix function equation of the equivalent circuit can be expressed as

(4) $$\left[\begin{array}{c} U_{I}\\ 0\\ 0 \end{array}\right]=\left[\begin{array}{c} Z_{0}+Z_{1}\\ -Z_{1}\\ 0 \end{array}\right.\begin{array}{c} -Z_{1}\\ Z_{1}+Z_{T}\\ -Z_{M} \end{array}\left.\begin{array}{c} 0\\ -Z_{M}\\ Z_{R}+R_{O} \end{array}\right]\left[\begin{array}{c} I_{I}\\ I_{T}\\ I_{O} \end{array}\right]$$

where $I_{T}$, $I_{T}$ and $I_{O}$ are the current phasors flowing through each loop, respectively. In addition, $Z_{0}$, $Z_{1}$, $Z_{T}$, $Z_{R}$ and $Z_{M}$, respectively, represent the equivalent impedances of transmitter-side series compensation capacitance $C_{0}$, transmitter-side parallel compensation inductance $L_{1}$, transmitter-side coil $L_{T}$ and compensation capacitance $C_{T}$, receiver-side coil $L_{R}$, and mutual inductance *M*, which can be expressed as

(5) $$\left\{\begin{array}{c} Z_{0}=jX_{0}=-j\frac{1}{\omega C_{0}}\\ Z_{1}=R_{1}+jX_{1}=R_{1}+j\omega L_{1}\\ Z_{T}=R_{T}+jX_{T}=R_{T}+j\left(\omega L_{T}-\frac{1}{\omega C_{T}}\right)\\ Z_{R}=R_{R}+jX_{R}=R_{R}+j\omega L_{R}\\ Z_{M}=jX_{M}=j\omega M \end{array}\right.$$

where $X_{0}$, $X_{1}$, $X_{T}$, $X_{R}$ and $X_{M}$ in Equation (5) represent the imaginary part of $Z_{0}$, $Z_{1}$, $Z_{T}$, $Z_{R}$ and $Z_{M}$, respectively. In addition, the coils in this study are all made of Lize-wire with extremely low internal resistance, $R_{1}$, $R_{T}$ and $R_{R}$ are therefore neglected in the circuit analysis. Then, substituting Equation (5) into (4), the current phasors in each loop can be deduced as

(6) $$\left\{\begin{array}{c} I_{I}=U_{I}\frac{jR{}_{O}\left(X_{1}+X_{T}\right)-X_{R}\left(X_{1}+X_{T}\right)+{X_{M}}^{2}}{AR_{O}+jB}\\ I_{T}=U_{I}\frac{jX_{1}\left(R{}_{O}+jX_{R}\right)}{AR_{O}+jB}\\ I_{O}=-U_{I}\frac{X_{1}X_{M}}{AR_{O}+jB} \end{array}\right..$$

The symbols *A* and *B*, which are introduced in Equation (6) for simplify, are expressed as

(7) $$\left\{\begin{array}{c} A=-(X_{0}X_{1}+X_{0}X_{T}+X_{1}X_{T})\\ B={X_{M}}^{2}\left(X_{1}+X_{0}\right)-X_{R}\left(X_{0}X_{1}+X_{0}X_{T}+X_{1}X_{T}\right) \end{array}\right.$$

### 2.2. Analysis of the CC Output Characteristic and ZPA Operation

To obtain the CC characteristic of the proposed system, $I_{O}$ should be independent of the AC equivalent resistance $R_{O}$. From Equation (6), the CC output can be achieved by making *A* equal to zero. The conditional equation that satisfies the CC characteristic can be expressed as

(8) $$X_{0}X_{1}+X_{0}X_{T}+X_{1}X_{T}=0$$

In addition, according to Equation (6) and $Z_{\mathrm{in}}$ = $U_{I}/I_{I}$, the system input impedance $Z_{\mathrm{in}}$ can be exported as Equation (9).

(9) $$Z_{\mathrm{in}}=\frac{-R_{O}\left(X_{0}X_{1}+X_{0}X_{T}+X_{1}X_{T}\right)+j{X_{M}}^{2}\left(X_{1}+X_{0}\right)-X_{R}\left(X_{0}X_{1}+X_{0}X_{T}+X_{1}X_{T}\right)}{{X_{M}}^{2}-X_{R}\left(X_{1}+X_{T}\right)+jR_{O}\left(X_{1}+X_{T}\right)}$$

To avoid the system losses caused by reactive power circulating current, the system should always run at ZPA operation. Specifically, $Z_{\mathrm{in}}$ should be purely resistive at the operating angular frequency ω. Then, the conditional equation that satisfies the ZPA operation of the system can be given as

(10) $${X_{M}}^{2}-X_{R}\left(X_{1}+X_{T}\right)=0$$

Based on Equations (8) and (10), $Z_{\mathrm{in}}$ is further deduced as

(11) $$Z_{\mathrm{in}}=\frac{{X_{M}}^{2}\left(X_{1}+X_{0}\right)}{R_{O}\left(X_{1}+X_{T}\right)}$$

Substituting Equations (8) and (10) into Equation (6), the RMS values of current phasors corresponding to each loop can be further simplified as

(12) $$\left\{\begin{array}{c} I_{I}=\frac{U_{I}R{}_{O}\left(X_{1}+X_{T}\right)}{{X_{M}}^{2}\left(X_{0}+X_{1}\right)}\\ I_{T}=\frac{U_{I}X_{1}\sqrt{R{{}_{O}}^{2}+{X_{R}}^{2}}}{{X_{M}}^{2}\left(X_{0}+X_{1}\right)}\\ I_{O}=\frac{U_{I}X_{1}}{X_{M}\left(X_{0}+X_{1}\right)} \end{array}\right.$$

According to Equation (12), the trans-conductance $G_{ui}$ can be deduced as

(13) $$G_{ui}=\frac{I_{O}}{U_{I}}=\frac{X_{1}}{X_{M}\left(X_{0}+X_{1}\right)}$$

Obviously, $Z_{in}$ in Equation (11) has only the real part, which proves that the system can achieve ZPA operation. Moreover, from Equation (12), only $I_{I}$ and $I_{T}$ are affected by the AC equivalent resistance $R_{O}$, while the $I_{O}$ is independent of $R_{O}$, which means that the system can achieve load-independent CC output. Therefore, through the reasonable compensation parameters design, the proposed CLC/N compensated WPT system can realize CC characteristics and ZPA operation.

## 3. Parameters Design and Simulation Verification

### 3.1. Calculation of the Compensation Components

Once the LCT structure is determined, the self-inductances $L_{R}$, $L_{T}$ and mutual inductance *M* are kept constant. Therefore, the CC output and ZPA operation of the proposed system depend on the transmitter-side parallel compensation inductance $L_{1}$ and transmitter-side series compensation capacitances $C_{0}$ and $C_{T}$. By solving the combined equations of (8), (10) and (13), $X_{0}$, $X_{1}$ and $X_{T}$ can be obtained as

(14) $$\left\{\begin{array}{c} X_{0}=\frac{-(G_{\mathrm{ui}}X_{M}-1)}{\omega L_{R}{G_{\mathrm{ui}}}^{2}}\\ X_{1}=\frac{X_{M}}{\omega L_{R}G_{\mathrm{ui}}}\\ X_{T}=\frac{G_{\mathrm{ui}}{X_{M}}^{2}-X_{M}}{\omega L_{R}G_{\mathrm{ui}}} \end{array}\right.$$

Substituting Equation (14) into (5), $C_{0}$, $L_{1}$ and $C_{T}$ are deduced as

(15) $$\left\{\begin{array}{c} C_{0}=\frac{L_{R}{G_{\mathrm{ui}}}^{2}}{\omega MG_{\mathrm{ui}}-1}\\ L_{1}=\frac{M}{\omega L_{T}G_{\mathrm{ui}}}\\ C_{T}=\frac{1}{\omega^{2}L_{T}+\frac{\omega M(1-\omega MG_{\mathrm{ui}})}{L_{R}G_{ui}}} \end{array}\right.$$

From Equation (15), the compensation inductance $L_{1}$ and compensation capacitances $C_{0}$ and $C_{T}$ are related to the trans-conductance $G_{\mathrm{ui}}$, which means that the different CC outputs in various application scenarios can be achieved by matching appropriate compensation components without replacing the LCT. The above descriptions strongly demonstrate that the proposed CLC/N compensated WPT system can achieve CC output that is unconstrained by the LCT parameters.

### 3.2. Design of the LCT and Determination of Circuit Parameters

**Figure 3:**
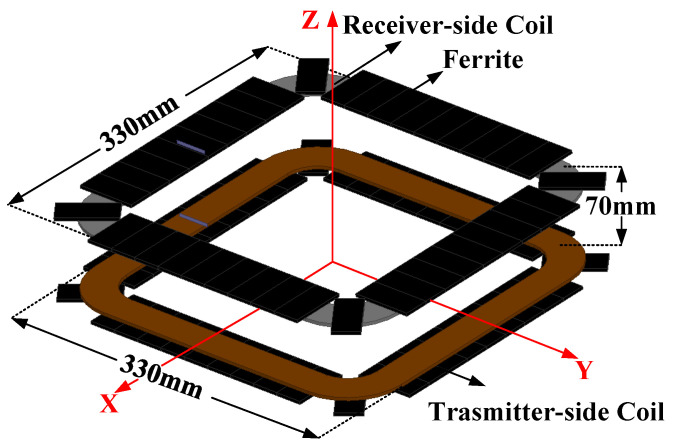
The 3D magnetic field model of the LCT.

The rounded square coil, which has the common advantages of a square coil and round coil, is employed for the LCT design. In addition, the transmitter-side coil and receiver-side coil are placed coaxially for enhancing coupling. Ferrite (PC 40) is utilized to improve the magnetic flux. The detailed specifications of the LCT are provided in Table 1. The magnetic field model of the LCT is simulated by finite element analysis (FEA) software 3D-ANSYS Maxwell, as shown in Figure 3.

In this study, the operating frequency and DC input voltage of the proposed CLC/N compensated WPT system is pre-set to 100 kHz and 20 V, respectively. The corresponding self-inductances and mutual inductance can be measured by using the LCR meter with sufficient accuracy, and $C_{0}$, $L_{1}$, $C_{T}$ can be calculated by Equation (15). The detailed circuit parameters of the CLC/N compensated WPT system are listed in Table 2.

### 3.3. Verification of the CC Characteristic and ZPA Operation

**Figure 4:**
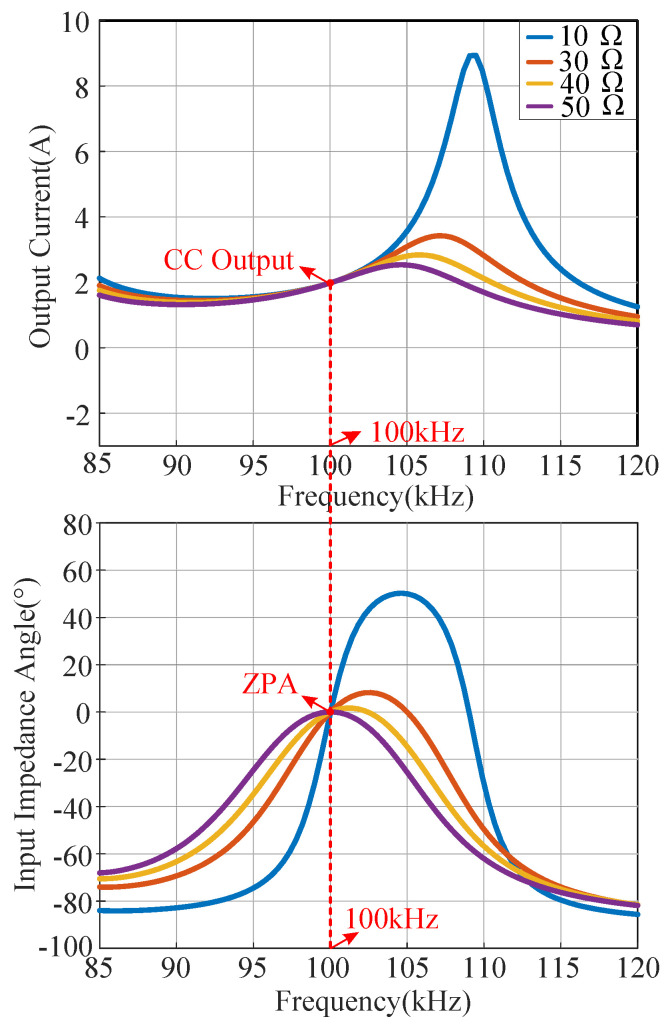
The sweep frequency curves of the output current and input impedance angle of the system under different load resistances.

According to the circuit parameters listed in Table 2, the sweep frequency curves of the output current and input impedance angle of the system under different load resistances are drawn, as shown in Figure 4. It can be seen that the proposed CLC/N compensated WPT system can realize both CC output and ZPA operation at the frequency point of 100 kHz, which proves the correctness of the theoretical analysis.

### 3.4. Verification of ZVS Operation

Since the MOSFETs in HFI has non-negligible parasitic capacitance, it is necessary to make the input impedance of the system slightly inductive to neutralize the charge of the capacitance in the MOSFETs, thereby realizing the ZVS operation and further improve the efficiency of the HFI. Based on Equation (9), the expression of $Z_{\mathrm{in}}$ is further simplified as

(16) $$Z_{\mathrm{in}}=\frac{AC+BD+j(BC-AD)}{C^{2}-D^{2}}$$

To facilitate calculation, four symbols A,B,C,D are introduced in Equation (16). The expressions of *A* and *B* are shown in Equation (7), and the mathematical expressions of *C* and *D* are shown in Equation (17).

(17) $$\left\{\begin{array}{c} C={X_{M}}^{2}-X_{R}\left(X_{1}+X_{T}\right)\\ D=R_{O}\left(X_{1}+X_{T}\right) \end{array}\right.$$

Based on Equations (16) and (17), the input impedance angle $\rho_{\mathrm{in}}$ of the proposed system is derived as

(18) $$\rho_{\mathrm{in}}=\arctan(\frac{BC-AD}{AC+BD})$$

**Figure 5:**
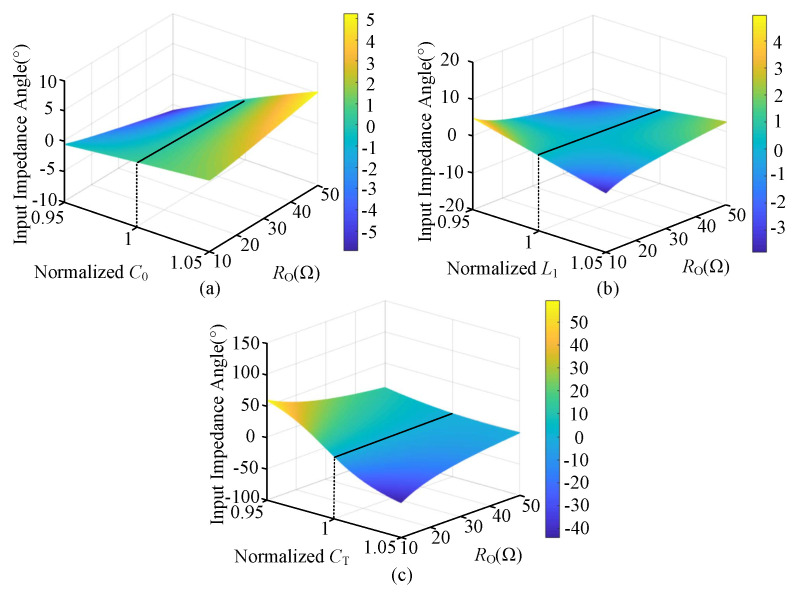
The input impedance angle versus different $R_{O}$ and normalized (**a**) $C_{0}$, (**b**) $L_{1}$ and (**c**) $C_{T}$.

**Figure 6:**
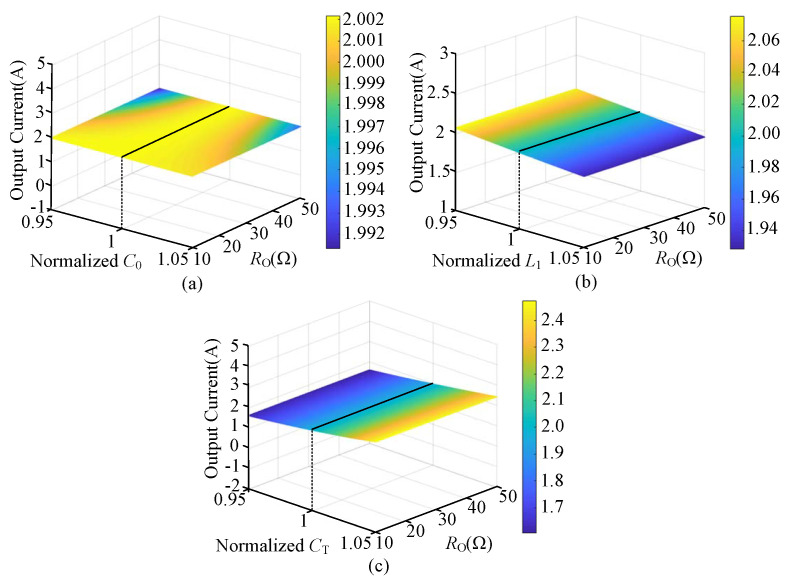
Output current versus different $R_{O}$ and normalized (**a**) $C_{0}$, (**b**) $L_{1}$ and (**c**) $C_{T}$.

It is worth noting that once the LCT structure is determined, $L_{T},L_{R}$ and *M* are fixed values, whereby the input impedance angle can be changed by adjusting the values of the compensation components $C_{0}$, $L_{1}$ and $C_{T}$. The input impedance angle versus variables $C_{0},L_{1}$ and $C_{T}$ at different load resistances are plotted, as shown in Figure 5. In order to more intuitively analyze the relationship between the compensation components and the input impedance angle, the normalized $C_{0},L_{1},C_{T}$ are adopted. It is clear that the normalized $L_{1}$ and normalized $C_{T}$ are negatively correlated with the input impedance angle $\rho_{\mathrm{in}}$, while the normalized $C_{0}$ is positively correlated with $\rho_{\mathrm{in}}$, thus the ZVS operation of the system can be achieved by decreasing the values of $L_{1}$ and $C_{T}$ or increasing the value of $C_{0}$. However, the CC output of the system is affected by the values of the compensation components, it is therefore necessary to analyze and select the ZVS operation scheme that has the least impact on the CC characteristic. Based on the designed parameters in Table 2, the relationship between the output current and the variables $C_{0},L_{1},C_{T}$ at different load resistances is analyzed, and the corresponding curves are shown in Figure 6.

As evident from Figure 6, the normalized $C_{0}$, $L_{1}$ and $C_{T}$ have different effects on the output current of the system. The change of normalized $C_{0}$ has almost no effect on the output current, while the changes of normalized $L_{1}$ and $C_{T}$ have more or less influence on the output current. Specifically, when the normalized $C_{0}$ is adjusted by ±5%, the output current is almost stable at 2 A and remains unchanged. When the normalized $L_{1}$ is adjusted ±5%, the change rate of output current is between ±3%. When the normalized $C_{T}$ is adjusted by ±5%, the change rate of output current is within ±20%. The above verification results show that the output current has the lowest sensitivity to the compensation capacitance $C_{0}$. Therefore, by adjusting the value of $C_{0}$, the ZVS operation can be achieved without affecting the CC output characteristic of the system. In addition, the above analysis results have reference significance for the selection of compensation components. For the CLC/N compensated CC-type WPT system introduced in this manuscript, the relatively low-precision compensation capacitance $C_{0}$ can be selected, thereby saving the hardware cost.

## 4. Comparison Analysis in Terms of Efficiency and Misalignment Performance

### 4.1. Efficiency of the CLC/N Topology

For a newly developed WPT system, power transfer efficiency is a key performance indicator. Taking $R_{1},R_{T}$ and $R_{R}$ into consideration, the efficiency of the proposed CLC/N topology can be expressed as

(19) $$\eta_{\mathrm{CLC}/N}=\frac{P_{\mathrm{OUT}}}{P_{\mathrm{IN}}}=\frac{P_{R_{O}}}{P_{R_{O}}+P_{R_{1}}+P_{R_{T}}+P_{R_{R}}}$$

$P_{\mathrm{OUT}}$ is the output power, which generally refers to the power consumed by the AC equivalent resistance, that is, $P_{R_{O}}$. $P_{\mathrm{IN}}$ is the input power, including the power $P_{R_{1}}$ consumed on the transmitter-side parallel compensation inductor, the power $P_{R_{T}}$ consumed on the transmitter-side coil, the power $P_{R_{R}}$ consumed on the receiver-side coil, and output power $P_{R_{O}}$. According to Equation (12), the corresponding power expressions can be derived as

(20) $$\left\{\begin{array}{c} P_{R_{O}}={I_{O}}^{2}R_{O}=\frac{R_{O}{\left(U_{I}X_{1}\right)}^{2}}{{X_{M}}^{2}{\left(X_{0}+X_{1}\right)}^{2}}\\ P_{R_{1}}={\left(I_{I}-I_{T}\right)}^{2}R_{1}=\frac{R_{1}{U_{I}}^{2}{\left[R_{O}\left(X_{1}+X_{T}\right)-X_{1}\sqrt{{R_{O}}^{2}+{X_{R}}^{2}}\right]}^{2}}{{X_{M}}^{4}{\left(X_{0}+X_{1}\right)}^{2}}\\ P_{R_{T}}={I_{T}}^{2}R_{T}=\frac{R_{T}{\left(U_{I}X_{1}\right)}^{2}\left({R_{O}}^{2}+{X_{R}}^{2}\right)}{{X_{M}}^{4}{\left(X_{0}+X_{1}\right)}^{2}}\\ P_{R_{R}}={I_{O}}^{2}R_{R}=\frac{R_{R}{\left(U_{I}X_{1}\right)}^{2}}{{X_{M}}^{2}{\left(X_{0}+X_{1}\right)}^{2}} \end{array}\right.$$

Substituting Equation (20) into (19), the efficiency expression of the CLC/N topology is derived in Equation (21).

(21) $$\begin{array}{c} \eta_{\mathrm{CLC}/N}=\frac{1}{1+R_{O}\frac{\left(R_{1}+R_{T}\right){X_{1}}^{2}+R_{1}{\left(X_{1}+X_{T}\right)}^{2}}{{\left(X_{1}X_{M}\right)}^{2}}+\frac{\left(R_{1}+R_{T}\right){X_{R}}^{2}+R_{R}{X_{M}}^{2}}{R_{O}{X_{M}}^{2}}-\frac{2R_{1}\left(X_{1}+X_{T}\right)\sqrt{{R_{O}}^{2}+{X_{R}}^{2}}}{X_{1}{X_{M}}^{2}}} \end{array}$$

### 4.2. Efficiency of the CLC/S Topology

**Figure 7:**
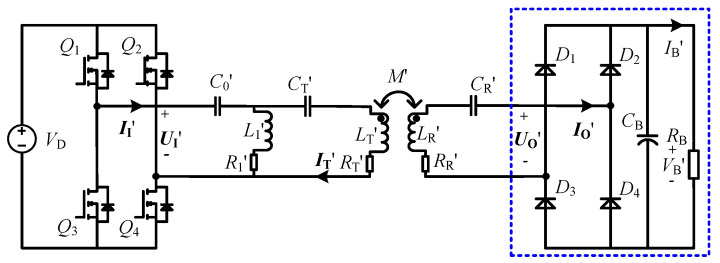
Architecture diagram of the CLC/S compensated WPT system.

In order to reflect the superiority of the proposed CLC/N compensated WPT system, the CLC/S compensated CC-Type WPT system is introduced for efficiency comparison. Figure 7 shows the architecture diagram of the CLC/S compensated WPT system, which mainly includes the DC input voltage source $V_{D}$, the HFI, the CLC/S topology, the rectifier, and the battery load $R_{B}$. The CLC/S topology includes transmitter-side series compensation capacitance ${C_{0}}^{\prime }$, transmitter-side parallel compensation inductance ${L_{1}}^{\prime }$, transmitter-side compensation capacitance ${C_{T}}^{\prime }$, transmitter-side coil ${L_{T}}^{\prime }$, receiver-side coil ${L_{R}}^{\prime }$, receiver-side compensation capacitance ${C_{R}}^{\prime }$. $M^{\prime }$ is the mutual inductance between ${L_{T}}^{\prime }$ and ${L_{R}}^{\prime }$. ${R_{1}}^{\prime }$, ${R_{T}}^{\prime }$ and ${R_{R}}^{\prime }$ are the parasitic resistances of ${L_{1}}^{\prime }$, ${L_{T}}^{\prime }$ and ${L_{R}}^{\prime }$, respectively.

**Figure 8:**
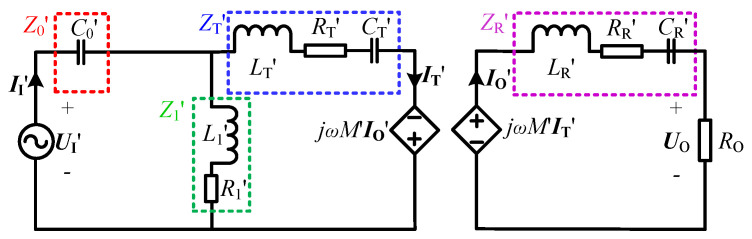
Equivalent circuit diagram of the CLC/S compensated WPT system.

To facilitate analysis, the equivalent circuit diagram of the CLC/S compensated WPT system is drawn, as shown in Figure 8. ${I_{I}}^{\prime }$, ${I_{T}}^{\prime }$ and ${I_{O}}^{\prime }$ represent the current phasors of each loop, respectively. Besides, ${U_{I}}^{\prime }$ and ${U_{O}}^{\prime }$ indicate the AC input voltage and the AC output voltage of the CLC/S topology, respectively.

According to KVL, the matrix function equation of the equivalent circuit can be expressed as

(22) $$\left[\begin{array}{c} {U_{I}}^{\prime }\\ 0\\ 0 \end{array}\right]=\left[\begin{array}{c} {Z_{0}}^{\prime }+{Z_{1}}^{\prime }\\ -{Z_{1}}^{\prime }\\ 0 \end{array}\right.\begin{array}{c} -{Z_{1}}^{\prime }\\ {Z_{1}}^{\prime }+{Z_{T}}^{\prime }\\ -{Z_{M}}^{\prime } \end{array}\left.\begin{array}{c} 0\\ -{Z_{M}}^{\prime }\\ {Z_{R}}^{\prime }+R_{O} \end{array}\right]\left[\begin{array}{c} {I_{I}}^{\prime }\\ {I_{T}}^{\prime }\\ {I_{O}}^{\prime } \end{array}\right]$$

where ${Z_{0}}^{\prime }$, ${Z_{1}}^{\prime }$, ${Z_{T}}^{\prime }$, ${Z_{R}}^{\prime }$ and ${Z_{M}}^{\prime }$ represent the equivalent impedances of ${C_{0}}^{\prime }$, ${L_{1}}^{\prime }$, ${L_{T}}^{\prime }$ and ${C_{T}}^{\prime }$, ${L_{R}}^{\prime }$ and ${C_{R}}^{\prime }$, and $M^{\prime }$, respectively. Furthermore, similar to the analysis of the proposed CLC/N compensated WPT system, when Equation (23) holds, the CLC/S compensated WPT system can achieve CC characteristics and ZPA operation.

(23) $$\left\{\begin{array}{c} {X_{0}}^{\prime }{X_{1}}^{\prime }+{X_{0}}^{\prime }{X_{T}}^{\prime }+{X_{1}}^{\prime }X_{T}{}^{\prime }=0\\ {{X_{M}}^{\prime }}^{2}-{X_{R}}^{\prime }\left({X_{1}}^{\prime }+{X_{T}}^{\prime }\right)=0 \end{array}\right.$$

where ${X_{0}}^{\prime }$, ${X_{1}}^{\prime }$, ${X_{T}}^{\prime }$, ${X_{R}}^{\prime }$ and ${X_{M}}^{\prime }$ represent the imaginary parts of ${Z_{0}}^{\prime }$, ${Z_{1}}^{\prime }$, ${Z_{T}}^{\prime }$, ${Z_{R}}^{\prime }$ and ${Z_{M}}^{\prime }$, respectively. According to Equations (22) and (23), the RMS values of ${I_{I}}^{\prime }$, ${I_{T}}^{\prime }$ and ${I_{O}}^{\prime }$ can be derived as

(24) $$\left\{\begin{array}{c} {I_{I}}^{\prime }=\frac{{U_{I}}^{\prime }R{}_{O}\left({X_{1}}^{\prime }+{X_{T}}^{\prime }\right)}{{{X_{M}}^{\prime }}^{2}\left({X_{0}}^{\prime }+{X_{1}}^{\prime }\right)}\\ {I_{T}}^{\prime }=\frac{{U_{I}}^{\prime }{X_{1}}^{\prime }\sqrt{R{{}_{O}}^{2}+{{X_{R}}^{\prime }}^{2}}}{{{X_{M}}^{\prime }}^{2}\left({X_{0}}^{\prime }+{X_{1}}^{\prime }\right)}\\ {I_{O}}^{\prime }=\frac{{U_{I}}^{\prime }{X_{1}}^{\prime }}{{X_{M}}^{\prime }\left({X_{0}}^{\prime }+{X_{1}}^{\prime }\right)} \end{array}\right.$$

Similar to Equation (21), the efficiency expression of the CLC/S topology can be calculated as Equation (25).

(25) $$\eta_{\mathrm{CLC}/S}=\frac{1}{1+R_{O}\frac{\left({R_{1}}^{\prime }+{R_{T}}^{\prime }\right){{X_{1}}^{\prime }}^{2}+{R_{1}}^{\prime }{\left({X_{1}}^{\prime }+{X_{T}}^{\prime }\right)}^{2}}{{\left({X_{1}}^{\prime }{X_{M}}^{\prime }\right)}^{2}}+\frac{\left({R_{1}}^{\prime }+{R_{T}}^{\prime }\right){{X_{R}}^{\prime }}^{2}+{R_{R}}^{\prime }{{X_{M}}^{\prime }}^{2}}{R_{O}{{X_{M}}^{\prime }}^{2}}-\frac{2{R_{1}}^{\prime }\left({X_{1}}^{\prime }+{X_{T}}^{\prime }\right)\sqrt{{R_{O}}^{2}+{{X_{R}}^{\prime }}^{2}}}{{X_{1}}^{\prime }{{X_{M}}^{\prime }}^{2}}}$$

### 4.3. Efficiency Comparison

To ensure the fairness of subsequent comparisons, the principles listed below should be followed:

(1) The output current of the two systems should be consistent to ensure the same functional characteristics.
(2) The load resistance of the two systems should be the same to maintain consistent output power.
(3) The same LCT structure and transmitter-side compensation inductor should be adopted in the two systems to ensure consistent internal resistances caused by Lize-wire.
(4) The operating frequency of the two systems should be equal.

**Figure 9:**
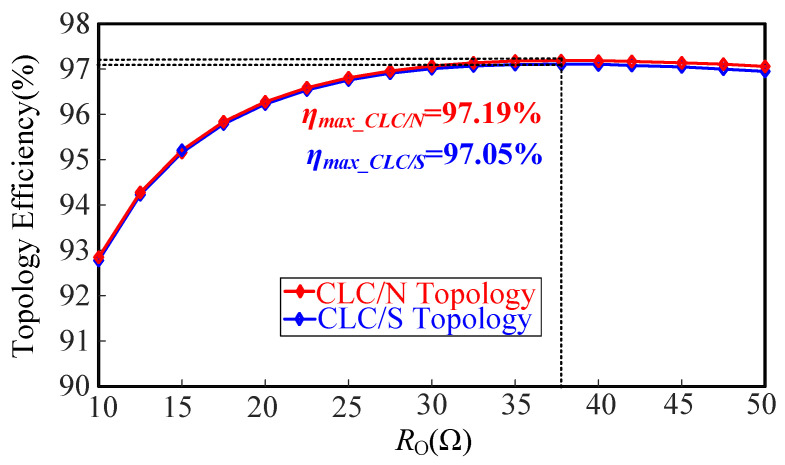
The efficiency profiles of the proposed CLC/N topology and CLC/S topology.

According to the above comparison principles and combining Equation (24), the circuit parameters of the CLC/S compensated WPT system are calculated and listed in Table 3. Then, substituting the circuit parameters in Table 2 into Equation (21) and the circuit parameters in Table 3 into Equation (25), the efficiency profiles of the proposed CLC/N topology and CLC/S topology are drawn, as shown in Figure 9.

As evident from Figure 9, the efficiency of the proposed CLC/N topology is slightly higher than that of the CLC/S topology in the full load range. In addition, it is worth mentioning that compared with the CLC/S compensated WPT system, the proposed CLC/N compensated WPT system has no compensation capacitor on the receiver. Therefore, the proposed CLC/N compensated WPT system is superior to the CLC/S compensated WPT system in terms of power transfer efficiency, compact receiver, and low cost.

## 5. Experiment Verification and Discussion

**Figure 10:**
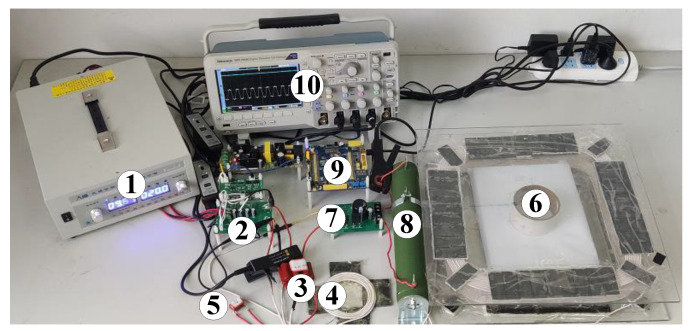
The experimental prototype of the CLC/N compensated WPT system.

To verify the correctness and rationality of the proposed CLC/N compensated WPT system, a confirmatory experimental prototype with CC output of 2 A is fabricated, as shown in Figure 10. It mainly includes DC input voltage source $V_{D}$, HFI, transmitter-side series compensation capacitor $C_{0}$, transmitter-side parallel compensation inductor $L_{1}$, compensation capacitor $C_{T}$ of the transmitter-side coil, LCT, rectifier, load $R_{B}$, controller and oscilloscope, which are numbered 1–10, respectively. The proposed CLC/N compensated WPT system is able to achieve CCO characteristics through its inherent structural properties, sophisticated control techniques are not required. Therefore, on the premise that the DC input voltage $V_{D}$ is constant, a simple microcontroller chip can be selected as the controller to regulate the output voltage of the inverter constant with 50% duty cycle. The specification types of the experimental components are listed in Table 4, and the detailed experimental circuit parameters of the proposed CLC/N compensated WPT system are provided in Table 5.

**Figure 11:**
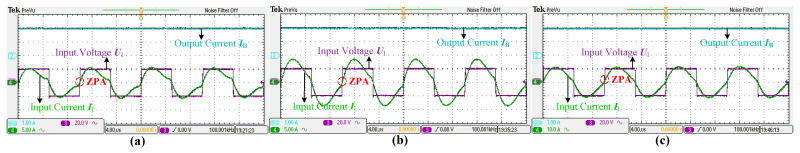
Experimental waveforms of $U_{I}$, $I_{I}$ and $I_{B}$ under ZPA condition when $R_{B}$ are (**a**) 15 Ω, (**b**) 25 Ω and (**c**) 35 Ω, respectively.

Firstly, the CC characteristic and ZPA operation of the proposed CLC/N compensated WPT system is validated. The corresponding waveforms of charging current $I_{B}$ and the output current/voltage ($I_{I}$/$U_{I}$) phasors of the HFI at the load resistances of 15 Ω, 25 Ω and 35 Ω are shown in Figure 11. It can be clearly seen that $I_{I}$ and $U_{I}$ always remain in phase, which means that the proposed system achieves the purely resistive input, namely ZPA operation. Moreover, the output current is maintained at 2 A under different load conditions, which proves that the proposed system can realize the load-independent CC characteristic.

**Figure 12:**
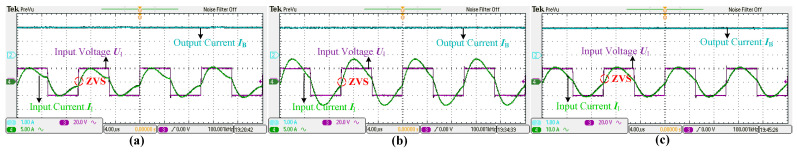
Experimental waveforms of $U_{I}$, $I_{I}$ and $I_{B}$ under ZVS condition when $R_{B}$ are (**a**) 15 Ω, (**b**) 25 Ω and (**c**) 35 Ω, respectively.

Then, the transmitter-side series compensation capacitance $C_{0\_\mathrm{ZPA}}$ is adjusted to $C_{0\_\mathrm{ZVS}}$ to validate the ZVS operation of the proposed system. The experimental waveforms of $U_{I}$, $I_{I}$ and $I_{B}$ under different load resistances of 15 Ω, 25 Ω and 35 Ω are shown Figure 12. It can be seen that $U_{I}$ slightly leads $I_{I}$, which indicates that ZVS operation of the proposed system can be achieved by slightly increasing the transmitter-side series compensation capacitance. In addition, it can be noticed that the output current $I_{B}$ remains at 2 A when $C_{0\_\mathrm{ZVS}}$ is adopted, which means that the implementation of ZVS operation has little effect on the CC output characteristic of the proposed system.

**Figure 13:**
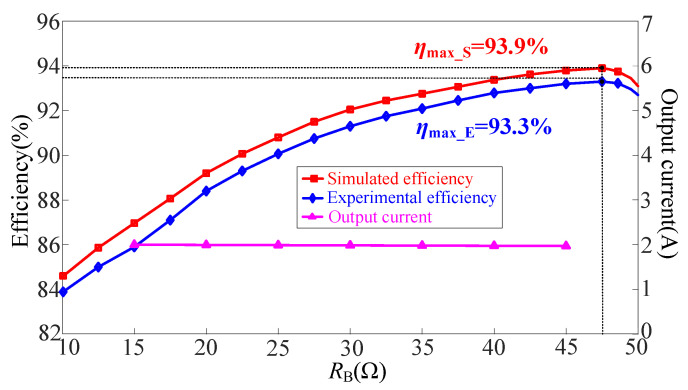
Profiles of power transfer efficiency and output current of the CLC/N compensated WPT system against varying load resistance.

Figure 13 demonstrates the power transfer efficiency and output current of the CLC/N compensated WPT system against varying load resistance, where max_S and max_E represent the maximum efficiency in simulation and experiment, respectively. It is noted from Figure 13 that the simulated efficiency is slightly higher than the experimental efficiency, and their efficiency peaks are 93.9% and 93.3%, respectively. However, the change trend of these two efficiency curves is the same, and both achieve high efficiency in the full load range. In addition, the output current of the CLC/N compensated WPT system is basically maintained at the set value of 2 A in the whole load range, which again verifies that the proposed system can achieve load-independent CC output characteristic.

To further embody the advantages of the proposed CLC/N compensated WPT system, several typical CC-type WPT systems are introduced for performance comparison, and the comparison results are shown in Table 6. Then, the following conclusions can be drawn:

(1) The output current of the proposed CLC/N compensated WPT system is not constrained by the LCT parameters, which means that the proposal in this study is functionally superior to the SS compensated WPT system in [22].
(2) The proposed CLC/N compensated WPT system has no bulky filter inductor behind the rectifier and no compensation components on the receiver, which not only saves the development cost but also ensures the compactness of the receiver of the system. These are the structural and economic advantages of the proposed CLC/N compensated WPT system compared to the CC-type WPT systems proposed in [23,24,25,26,27,28,29].

## 6. Conclusions

This manuscript proposes a new CLC/N compensated CC-type WPT system and the corresponding parameter design method, which can achieve the load-independent CC output function and ZPA operation. The proposed system can obtain constant output current unconstrained by the LCT parameters, thus improving compatibility. In addition, by slightly increasing the transmitter-side series compensation capacitance $C_{0}$, the system can achieve ZVS operation without affecting the CC characteristic, further ensuring the high efficiency of the WPT system. Moreover, there are no compensation components on the receiver and no bulky filter inductor behind the rectifier, which effectively saves hardware development cost and ensures the compactness of the receiver. Finally, the overall performance of the proposed CLC/N compensated WPT system is well verified by experiments. Overall, compared with the existing WPT systems based on various CC-type topologies, the proposed CLC/N compensated WPT system has obvious advantages in function, structure and economy, and has potential application value.

## Figures and Tables

**Table 1:** The size parameters of the LCT in the CLC/N compensated WPT system.

Parameter	Specifications	Coil Turns
Transmitter-side coil	2.8 mm × 2.8 mm	10
Receiver-side coil	2.8 mm × 2.8 mm	10
Air gap	70 mm	−−

**Table 2:** Theoretical circuit parameters of the proposed CLC/N compensated WPT system.

Parameters	Value	Parameters	Value
$V_{D}$	20 V	$I_{B}$	2 A
$L_{T}$	100 μH	$R_{T}$	0.12Ω
$L_{R}$	100 μH	$R_{R}$	0.12 Ω
$L_{1}$	5.85 μH	$R_{1}$	0.04 Ω
$C_{0}$	608.3 nF	*M*	45 μH
$C_{T}$	29.58 nF	*k*	0.45

**Table 3:** Theoretical circuit parameters of the CLC/S compensated WPT system.

Parameters	Value	Parameters	Value
$V_{D}$	20 V	${I_{B}}^{\prime }$	2 A
${L_{T}}^{\prime }$	100 μH	${R_{T}}^{\prime }$	0.12 Ω
${L_{R}}^{\prime }$	100 μH	${R_{R}}^{\prime }$	0.12 Ω
${L_{1}}^{\prime }$	5.85 μH	${R_{1}}^{\prime }$	0.04 Ω
${C_{0}}^{\prime }$	600.75 nF	$M^{\prime }$	45 μH
${C_{T}}^{\prime }$	29.81 nF	$k^{\prime }$	0.45
${C_{R}}^{\prime }$	821.41 nF	—	—

**Table 4:** The specification types of the experimental components.

Experimental Components	Specification Types
Diodes for rectifier	MBR16100CT
MOSFETs for HFI	IRFP250N
Controller	STM32F103-C8T6
Lize-wire	400 strands, 2.8 mm

**Table 5:** Experimental circuit parameters of the proposed CLC/N compensated WPT system.

Parameters	Value	Parameters	Value
Power rating	192 W	Voltage rating	96 V
$V_{D}$	20 V	$I_{B}$	2 A
$L_{T}$	100.3 μH	$R_{T}$	0.12 Ω
$L_{R}$	100.2 μH	$R_{R}$	0.12 Ω
$L_{1}$	5.91 μH	$R_{1}$	0.04 Ω
$C_{T}$	29.65 nF	*M*	45 μH
$C_{0\_\mathrm{ZPA}}$	610.3 nF	$C_{0\_\mathrm{ZVS}}$	640.5 nF
*k*	0.45	—	—

**Table 6:** Comparison results of this work with previous related studies.

Proposed in	Ref. [22]	Ref. [23]	Ref. [24]	Ref. [25]	Ref. [26]	Ref. [27]	Ref. [28]	Ref. [29]	This Work
CC-type topology	SS	LC/CC	LCL/P	LCC/LCC	Four-coil	LC/CL	Three-coil	LC/S	CLC/N
Number of Coils	2	2	2	2	4	2	3	2	2
Max power	15 W	50 W	100 W	6.6 kW	6.6 kW	96.8 W	225 W	63.8 W	192 W
Peak efficiency	92.6%	92.4%	92.8%	96.1%	96.3%	90.3%	92.5%	91.9%	93.3%
Number of transmitter-side compensation components	1	2	2	3	2	2	2	2	3
Number of receiver-side compensation components	1	2	1	3	2	2	1	1	0
Output current unconstrained by LCT parameters	No	Yes	Yes	Yes	Yes	Yes	Yes	Yes	Yes
Without filter inductor behind the rectifier	Yes	No	No	Yes	Yes	Yes	Yes	Yes	Yes
Compact and low-cost receiver	No	No	No	No	No	No	No	No	Yes

## Data Availability

Not applicable.
